# Editorial: Cuproptosis and tumor

**DOI:** 10.3389/fcell.2023.1307501

**Published:** 2023-11-24

**Authors:** Bing Liu, Jian-Nan Liu, Guo-Jun Chen, Chun Xu, Lin-Lin Bu

**Affiliations:** ^1^ State Key Laboratory of Oral and Maxillofacial Reconstruction and Regeneration, Key Laboratory of Oral Biomedicine Ministry of Education, Hubei Key Laboratory of Stomatology, School and Hospital of Stomatology, Wuhan University, Wuhan, Hubei, China; ^2^ Department of Oral and Maxillofacial—Head Neck Oncology, School and Hospital of Stomatology, Wuhan University, Wuhan, Hubei, China; ^3^ Department of Oral Maxillofacial—Head and Neck Oncology, Shanghai Ninth People’s Hospital Affiliated to Shanghai Jiao Tong University School of Medicine, Shanghai, China; ^4^ Department of Biomedical Engineering and Rosalind and Morris Goodman Cancer Institute, McGill University, Montreal, QC, Canada; ^5^ School of Dentistry, The University of Queensland, Brisbane, QLD, Australia

**Keywords:** cell death, cuproptosis, molecular mechanism of cuproptosis, tumor, drug delivery system

As a trace element, copper is widely involved in the physiological activities of cells and plays an important role. Accumulation of copper in cells can induce oxidative stress and disrupt cellular function, thus copper homeostasis in cells is strictly regulated. Cuproptosis is a new type of programmed cell death induced by copper and is different from other types such as apoptosis, pyroptosis, and ferroptosis (Tsvetkov, et al., 2022). Copper ions bind to lipoacyl proteins during the tricarboxylic acid (TCA) cycle, leading to abnormal oligomerization of lipoacyl protein (Li, et al., 2022). In addition, the level of iron-sulfur cluster proteins can be reduced by copper ions, resulting in toxic stress reactions in proteins and leading to cell death. Cuproptosis impacts the pathogenesis of various diseases, including hepatolenticular degeneration, neurodegenerative diseases, and cancer (Wang, et al., 2023). Therefore, targeting cuprotosis may become a potential treatment method for various diseases and has attracted widespread attention.

This Research Topic focuses on the molecular mechanism of cuprotosis in the development of tumors and the potential therapeutic approach to targeting cuprotosis. Based on the significant impact of cuproptosis in the pathogenesis of colorectal cancer, Li et al. identified potential cuprotosis-related genes (CRGs) and developed a new predictive model using LASSO regression and multivariate Cox stepwise regression in the TCGA dataset, which evaluates the immune characteristics of colorectal cancer patients while predicting their prognosis. In addition, Wang et al. comprehensively analyzed the relationship between CRG and TME in colon adenocarcinoma (COAD), constructed a CRG risk scoring system, and accurately predicted the survival rate of COAD patients. The CRG risk scoring systems have provided clinical doctors with new insights to develop more effective and personalized treatment strategies. Fan et al. designed a new nomograph containing CRG scores and clinical characteristics, which can predict the 3-year, 5-year, and 7-year recurrence risk of ER + breast cancer. Liu et al. revealed the potential impact on the overall survival period, immune invasion, drug sensitivity, and metabolic spectrum of breast cancer through CRG. Similarly, scholars have also explored the prognostic value of CRG in prostate adenocarcinoma, lung adenocarcinoma, and gastric cancer.

The impact of cuproptosis on the occurrence and development of hepatocellular carcinoma, as well as its potential targets and prognostic value, seems to have aroused great interest. For example, Shao et al. and Shi et al. developed scoring models based on CRG to predict the prognosis of hepatocellular carcinoma and revealed the potential synergistic effect of novel immunotherapies such as TIGHT, CD274, and LAG-3 on cuproptosis. Cao et al. explained the characteristics of cuproptosis in hepatocellular carcinoma through single-cell sequencing and genetic multiomics and identified that BEX1 may be a key hub gene mediating cuproptosis in hepatocellular carcinoma and serve as a potential therapeutic target. Wang et al. explained the potential role of targeted cuproptosis in targeted immune microenvironment therapy for hepatocellular carcinoma and proposed that CRG can serve as a biomarker for immune checkpoint inhibitor therapy.

Although this Research Topic has collected many interesting and valuable research results, the relationship between cuproptosis and tumors still needs to be further explored. A deeper understanding of the role of cuproptosis in different tumor mechanisms should be explored, which may include aspects such as cell death, energy metabolism, and tumor immunity (Chen, et al., 2022). In addition, the role of targeted cuproptosis in tumor treatment should also be taken seriously. Recent studies have shown that inducing abnormal programmed cell death may be a potential method for treating and preventing tumor diseases. Therefore, targeting cuproptosis to increase the level of cuproptosis in tumor cells provides a new approach for tumor treatment. Cuproptosis, as an immunogenic death (ICD), can promote the release of tumor antigens, increase antigen presentation levels, promote T cell activation, and enhance anti-tumor immunity (Xie, et al., 2023). Therefore, targeting cuproptosis as a supplement to immunotherapy or an adjuvant therapy to improve the effectiveness of immunotherapy has enormous potential application value. The combination of cuproptosis with other therapies such as chemotherapy, radiotherapy, and photodynamic therapy has also received attention (Li, et al., 2023). Currently, drug delivery systems have received a lot of attention. The drug delivery system can accurately deliver drugs that induce cuproptosis to the tumor microenvironment. While improving the level of cuproptosis in tumor cells, it can reduce the systemic toxicity and side effects of drugs, thereby improving the survival period and quality of life of tumor patients. This will be the focus of future research.
